# Validity and Reliability of Single-item Questions about Physical Activity

**DOI:** 10.2188/jea.11.211

**Published:** 2007-11-30

**Authors:** Nobuo Iwai, Shigeru Hisamichi, Norihiko Hayakawa, Yutaka Inaba, Tadashi Nagaoka, Hiroki Sugimori, Nao Seki, Kiyomi Sakata, Koji Suzuki, Akiko Tamakoshi, Yosikazu Nakamura, Akio Yamamoto, Yoshikazu Nishino, Atsushi Ogihara, Naoyuki Okamoto, Hiroshi Suzuki, Seiji Morioka, Yoshinori Ito, Kenji Wakai, Toshiyuki Ojima, Heizo Tanaka, Takayuki Nose, Yoshiyuki Ohno

**Affiliations:** 1 Department of Public Health, Faculty of Medicine, Tottori University.; 2 Division of Public Health, Department of Social Medicine, Tohoku University Graduate School of Medicine.; 3 Department of Epidemiology, Research Institute for Radiation Biology and Medicine, Hiroshima University.; 4 Department of Epidemiology and Environmental Health, Juntendo University School of Medicine.; 5 Department of Epidemiology, Research Institute, Kanagawa Cancer Center.; 6 Department of Preventive Medicine, St. Marianna University School of Medicine.; 7 Department of Public Health, Niigata University School of Medicine.; 8 Department of Public Health, Wakayama Medical College.; 9 Department of Public Health, Fujita Health University School of Health Sciences.; 10 Department of Preventive Medicine/Biostatistics and Medical Decision Making, Nagoya University Graduate School of Medicine.; 11 Department of Public Health, Jichi Medical School.; 12 Division of Epidemiology and Information, Hyogo Prefectural Institute of Public Health.; 13 Department of Epidemiology, Medical Research Institute, Tokyo Medical and Dental University.

**Keywords:** validity, reliability, physical activity, single-item question, lifestyle-related disease

## Abstract

The Japan Collaborative Cohort Study for Evaluation of Cancer Risk Sponsored by Monbusho (JACC Study) included in its self-administered questionnaires some single-item questions concerning physical activity. We examined the validity of the questions among 1,730 Japanese adults and the reliability of the questions among 1,075 Japanese adults. The validity of the sports and physical exercise questions was estimated by comparing the self-administered questionnaire responses with the time spent on the activity and the energy expenditure index for the previous 12-month period, elicited by the interviewing method used in the Japan Lifestyle Monitoring Study with a minor modification. The Spearman’s rank correlation coefficients ranged from 0.43 to 0.60, showing moderate correlations. On the other hand, test-retest reliability was estimated by comparing the responses from two separate surveys conducted roughly one year apart. Weighted kappa coefficients of sports and physical exercise questions, classified according to sex and age, ranged from 0.39 to 0.56, showing moderate reliability; and those of a question about walking ranged from 0.25 to 0.39, showing fair reliability. We suggest that measuring physical activity level with these single-item questions may be appropriate for establishing baseline data that reflects long-term physical activity in a large-scale cohort study targeting lifestyle-related diseases.

## INTRODUCTION

The health benefits of habitual physical activity have been reported by many epidemiological studies using physical activity questionnaires^1^^)^. Currently, the questionnaire is the most widely used measurement method for assessing physical activity in study populations, and various questionnaires have been developed^2^^)^. Many of them are complex, with several questions from which to estimate a subject’s energy expenditure from activity; the validity and reliability of these have been investigated^3^^-^^10^^)^. However, in practical use, single-item questions are often included in epidemiological and public health questionnaires designed to measure health status and exposure to risk factors^11^^)^. Measuring physical activity with a single-item question has the advantage of requiring less time and money to obtain the data. The simplicity is especially needed in a large-scale epidemiological study that uses a lengthy questionnaire to collect data on many factors, including physical activity. There are only a few reports on the validity and reliability of single-item questions about physical activity^12^^, ^^13^^)^. The Japan Collaborative Cohort Study for Evaluation of Cancer Risk Sponsored by Monbusho (JACC Study), a multicenter study in Japan begun in 1986^14^^)^, included some single-item questions concerning physical activity in its self-administered questionnaires given at baseline and interim surveys. However, these questions seemed to have limitations, so we investigated their validity and reliability among many Japanese adults.

## METHODS

### JACC questionnaires

The JACC questionnaires include the following 3 single-item questions and corresponding response sets:

*Question 1: How much time per week on average do you spend engaging in sports or physical exercise? 1) at least 5 hours 2) 3 - 4 hours 3) 1- 2 hours 4) little*
*Question 2: How much time per day on average do you spend walking indoors or outside? 1) more than 1 hour 2) 30 minutes - 1 hour 3) about 30 minutes 4) little*
*Question 3: How often did you engage in sports or physical exercise over the past year or two? 1) seldom 2) sometimes 3) about once a week 4) at least twice a week*

Question 1 and 2 were used in the baseline survey and Question 3 was used in the interim survey. The questions focus on usual leisure-time physical activity (Question 1 and 3) and daily walking patterns (Question 2).

These questions were thought to have the following limitations. First, the definitions of “sports” and “physical exercise” were not presented to the subjects beforehand and the types of activities they had engaged in were not investigated. The word “undou” was used as the Japanese translation of “physical exercise”, but “undou” means “a bodily movement” in another sense too, so there was the possibility of measurement error based on the subjects’ interpretations of the questions. Some of the subjects may have included occupation-related physical activity, such as farming chores, into “undou” (physical exercise) and some may have excluded activities of light intensity, such as walking for pleasure, and only included “vigorous sports competition” as physical exercise. In fact, in an investigation at one of the JACC study fields, when subjects who checked the first three responses to Question 1 were asked to list their activity, 8% of subjects responded with “farming” or “job” and the percentage of occupation-related answers was the highest among the subjects who answered that they were most active. In addition, it was not clear to what degree the responses to Question 1 or Question 3 correlated with the energy expended during the activity. Second, although leisure-time physical activity and walking were assumed to have seasonal variability^9^^)^, the JACC surveys were not necessarily conducted at the same season and the subjects may have responded by recalling their most recent activities and attributing that frequency to the entire year.

Therefore, we chose the interviewing method that was used to assess leisure-time physical activity in the Japan Lifestyle Monitoring Study^15^^, ^^16^^)^ as the criterion-measuring instrument and investigated the criterion validity of Questions 1 and 3, as well as the test-retest reliability of Questions 1-3, after a nearly 1-year interval.

### Subjects and study design

The first survey was conducted in 10 communities belonging to the JACC study fields, one neighboring community, and one company. In the communities, subjects were recruited from adults younger than 80 years old; in the company, subjects were recruited from employees younger than 40 years old who participated in a work site health check-up. Different fields were surveyed in different seasons, as shown in Table 1. Of 1,880 subjects who completed the first self-administered questionnaire (including Questions 1-3), 90% were participants in a health check-up (5 fields) or in epidemiological research (3 fields), 6% were involved in health education, and 4% were neighbors or family members of the researchers/interviewers. They were selected independent of whether they played sports.

**Table 1.** Group characteristics of 1,880 subjects and the survey periods.

Field No.	Region	Prefecture	N	Age (yr)			First survey month/year	Second survey month/year

				Mean	SD	Range		
1	Hokkaido	Hokkaido	867	59.8	10.1	39-79	8/1998	8/1999
2	Tohoku	Miyagi	105	63.0	8.5	46-77	8-9/1997	11-12/1998
3*	Kanto	Tochigi	131	56.0	8.9	40-70	11/1998	–
4	Kanto	Kanagawa	45	61.7	7.3	48-78	1-3/1998	3/1999
5	Koshinetu	Yamanashi	74	63.4	11.2	36-79	12/1997	1/1999
6	Koshinetu	Niigata	150	60.8	9.0	41-78	5/1998	5/1999
7*	Chubu	Gifu	58	56.4	11.1	25-70	8/1998	–
8	Kinki	Wakayama	80	65.5	9.3	40-79	7/1998	10/1999
9	Kinki	Hyougo	24	52.6	7.6	41-69	12/1998-1/1999	2/2000
10	Chugoku	Tottori	210	53.6	9.3	40-69	11/1996	11/1997
							10/1997	10/1998
							11/1998	11/1999
11	Chugoku	Hiroshima	39	68.3	5.8	52-77	11/1997	2/1999
12*	Company	Tokyo	97	30.3	4.7	22-39	5-6/1998	–

* Only validation study was conducted.

Of these 1880 subjects, 1730 were interviewed for the validation study, either on the same day or within 2 months after they had filled out the questionnaire. Furthermore, in 9 study fields, a second self-administered questionnaire survey was conducted after roughly one year (average, 12.6 months; range, 11.8-15.9 months). Of the 1880 subjects who completed first questionnaire, 1075 between the ages of 40 and 79 years participated in the second survey. Table 2 shows the distribution of subjects in the validation and reliability studies according to age grouping and sex. The subjects who participated in the interview or second survey were selected regardless of which response they checked in the first questionnaire survey.

**Table 2.** Distribution of subjects in the validation and reliability studies according to age group and sex.

Age (yr)	22-29	30-39	40-49	50-59	60-69	70-79	Total
	N(%)	N(%)	N(%)	N(%)	N(%)	N(%)	N(%)
Validation study							
Men	27 (3.7)	50 (6.8)	138 (18.7)	143 (19.4)	250 (33.8)	131 (17.7)	739 (100)
Women	15 (1.5)	29 (2.9)	184 (18.6)	253 (25.5)	357 (36.0)	153 (15.4)	991 (100)
Total	42 (2.4)	79 (4.6)	322 (18.6)	396 (22.9)	607 (35.1)	284 (16.4)	1730 (100)

Reliability study							
Men	–	–	85 (20.0)	84 (19.8)	160 (37.7)	96 (22.6)	425 (100)
Women	–	–	103 (15.9)	177 (27.2)	262 (40.3)	108 (16.6)	650 (100)
Total	–	–	188 (17.5)	261 (24.3)	422 (39.3)	204 (19.0)	1075 (100)

### Validation study of sports and physical exercise questions

Criterion validity was determined by comparing the responses to Question 1 or Question 3 in the first survey and the indices derived from the interviewing method. Participants were interviewed by research personnel trained to use the interviewing method that was used in the Japan Lifestyle Monitoring Study^16^^)^ with a minor modification. The interviewers asked subjects several questions about sports and physical exercise from an interview manual.

First, participants were asked if they had engaged in any sports or physical exercise in the 12 months prior to the interview. Those who answered yes were then asked what kind of activity they had engaged in; those who answered no were asked again if they had engaged in any light activities such as walking for pleasure or gathering wild vegetables in fields and forests. The activities mentioned by the participants that agreed with the definition of sports and physical exercise were then recorded on the questionnaire.

“Sports or physical exercise” was defined as organized competitive sports, recreational activities, or other leisure-time physical activities that are done for physical training, health promotion, or relaxation. For an activity to be included in the survey, the intensity of the activity needed to be greater than or equal to that of walking. Walking or cycling to and from work and household chores were excluded from the survey by our definition.

Second, participants were asked to estimate the frequency and average duration of each activity, in a format similar to that of the Minnesota leisure-time physical activity questionnaire^17^^)^. Questions on activity frequency for six 2-month periods were asked, but for convenient assessment, a 2-month period was considered to be 8 weeks. Questions on activity duration were meant to exclude time spent at rest.

Data obtained from all study fields were then checked by one of the authors (N. I.) to improve accuracy. According to the predetermined definition, activities which had an energy expenditure lower than walking or which were thought to be chores were excluded from analysis even if they were recorded on the questionnaires. In addition, gardening, some voluntary activities, and art activities were excluded because they were not thought to be sports or physical exercises. Finally, 32 miscellaneous activities from the records of 937 people who had engaged in some type of activity were excluded, yet these exclusions did not affect the validity of the questions.

The number of minutes spent on sports and physical exercise over a year were summed and summarized as the average time per week. The number of minutes spent on each activity in a year was multiplied by the activity’s intensity, estimated as a MET score (a multiple of the resting metabolic rate) of 2.5, 4.5, 6.5, or 8.5, resulting in a leisure-time physical activity score (METs·min/day) comparable to those in the Japan Lifestyle Monitoring Study.

The leisure-time physical activity score (LTPA score) roughly reflects the rate of energy expenditure per resting metabolic rate or body weight. The validity and reliability of the LTPA score in the Japan Lifestyle Monitoring Study are described elsewhere. In short, the LTPA score is inversely correlated with resting heart rate after adjusting for possible confounding factors (men, r = -0.08 and p<0.05; women, r = -0.10 and p<0.01)^16^^)^. The test-retest coefficient was 0.59 (p<0.001) after 1 month^18^^)^.

We calculated Spearman’s rank correlation coefficients by correlating the response to Question 1 or Question 3 with the time spent on sports and physical exercise and the LTPA scores that were derived from the interviewing method. We examined the distributions of the time spent on the activity derived from the interview by the response categories of Questions 1 and 3. The Kruskal-Wallis test was used to assess differences in the mean levels of activity time.

### Reliability study

Test-retest reliability was estimated by comparing the response categories from two separate surveys conducted almost one year apart. For calculation of reliability coefficients, scores of 5, 3.5, 1.5 and 0 were given to the response categories of Question 1; scores of 1, 0.75, 0.5 and 0 were given to those of Question 2; and scores of 0, 0.5, 1 and 2 were given to those of Question 3. These scorings are presented in Table 3. Then weighted kappa coefficients were calculated using linear weights^19^^, ^^20^^)^ proportional to the deviation of individual scores. The weighted kappa coefficient is recommended as an index of agreement for reporting the reliability of ordinal data in several categories^21^^)^. Assessment of reliability using weighted kappa coefficients was done according to the criteria presented by Landis and Koch^22^^)^.

**Table 3.** Distribution of the responses from 1,880 subjects to Questions 1-3 in the first survey by sex and age group.

Age (yr)		Men								Women					
	
		22-39			40-59			60-79		22-39		40-59		60-79	
		N(%)			N(%)			N(%)		N(%)		N(%)		N(%)	
Question 1 : time spent on sports and physical exercise															
little	(S=0)	38	( 49.4)		231		( 73.1)	247	( 61.0)	25	( 56.8)	332	( 69.0)	347	( 64.1)
1-2hr/week	(S=1.5)	25	( 32.5)		47		( 14.9)	64	( 15.8)	17	( 38.6)	78	( 16.2)	100	( 18.5)
3-4hr/week	(S=3.5)	10	( 13.0)		24		( 7.6)	35	( 8.6)	2	( 4.5)	39	( 8.1)	47	( 8.7)
>=5hr/week	(S=5)	4	( 5.2)		14		( 4.4)	59	( 14.6)	0		32	( 6.7)	47	( 8.7)
Total		77	(100.0)		316		(100.0)	405	(100.0)	44	(100.0)	481	(100.0)	541	(100.0)

Question 2 :daily walking time															
little	(S=0)	2	( 2.6)		49		( 15.8)	47	( 11.7)	2	( 4.8)	55	( 11.6)	49	( 9.3)
about 30min	(S=0.5)	22	( 28.6)		47		( 15.2)	62	( 15.4)	13	( 31.0)	79	( 16.7)	82	( 15.5)
30min-1hr	(S=0.75)	30	( 39.0)		67		( 21.6)	87	( 21.6)	13	( 31.0)	87	( 18.4)	130	(24.6)
>1hr	(S=1)	23	( 29.9)		147		( 47.4)	207	( 51.4)	14	( 33.3)	253	( 53.4)	268	( 50.7)
Total		77	(100.0)		310		(100.0)	403	(100.0)	42	(100.0)	474	(100.0)	529	(100.0)

Question 3 : frequency of sports and physical exercise															
seldom	(S=0)	19		( 24.7)	184	( 58.8)		227	( 57.8)	14	( 31.8)	310	( 65.4)	320	( 60.7)
sometimes	(S=0.5)	36		( 46.8)	83	( 26.5)		74	( 18.8)	14	( 31.8)	71	( 15.0)	83	( 15.7)
about once/week	(S=1)	12		( 15.6)	26	( 8.9)		22	( 5.6)	13	( 29.5)	41	( 8.6)	40	( 7.6)
>=twice /week	(S=2)	10		( 13.0)	18	( 5.8)		70	( 17.8)	3	( 6.8)	52	( 11.0)	84	( 15.9)
Total		77		(100.0)	313	(100.0)		393	(100.0)	44	(100.0)	474	(100.0)	527	(100.0)

S in parentheses: Scores used for calculating weighted kappa coefficients.

Total number of subjects varied by question because of missing values.

The interval between the two surveys in the reliability study ranged from 11.8 to 15.9 months. This interval was often greater than 12 months, and it was assumed that reliability was affected by seasonal variations. We divided the subjects into two subgroups, according to the length of this interval: those who were surveyed at an interval very close to 12 months (11.5-12.4 months), and those who were surveyed at an interval in the range of 12.5 to 15.9 months. We then calculated the reliability coefficients of these groups separately.

## RESULTS

Table 3 shows the distributions of responses from the first self-administered questionnaire by sex and age group. The distributions were biased toward inactivity in the sports and physical exercise questions and biased toward activity in the walking question.

Table 4 shows the Spearman’s rank correlation coefficients from correlating the rank scores of the single-item questions with the indices derived from the interview. The validity coefficients ranged from 0.43 to 0.60, showing moderate correlations. The coefficients of Question 1 were not calculated among women 22-39 years old because the sample size was so small that no sample was present in the most active category of Question 1. Men 40-59 years old had a slightly higher correlation coefficient than men 60-79 years old; a similar relationship was not observed in the women’s data.

**Table 4.** Spearman’s rank correlation coefficients from correlating the response to Question 1 or Question 3 with the activity time and LTPA score derived from the interview.

	All	Age group (yr)		

		22-39 #	40-59	60-79
Men	n=739	n=77	n=281	n=381
Age (yr) : Mean (SD)	57.5 (13.2)	31.3 (4.9)	50.3 (5.5)	68.1 (4.5)
Question 1 : time				
vs activity time	0.52	0.51	0.54	0.49
vs LTPA score	0.53	0.57	0.54	0.50
Question 3 : frequency				
vs activity time	0.51	0.43	0.57	0.47
vs LTPA score	0.53	0.48	0.58	0.47

Women	n=991	n=44	n=437	n=510
Age (yr) : Mean (SD)	58.4 (11.1)	32.2 (5.9)	50.7 (5.5)	67.3 (4.8)
Question 1 : time				
vs activity time	0.57	–	0.57	0.57
vs LTPA score	0.58	–	0.58	0.58
Question 3 : frequency				
vs activity time	0.58	0.47**	0.58	0.57
vs LTPA score	0.59	0.46*	0.60	0.58

All P-values of the correlation were 0.0001, except *P=0.002 and **P=0.001.

# The coefficients of Question 1 were not calculated for women because of small sample size.

Table 5 shows quartiles of time spent on sports and physical exercise, derived from the interview, for each of the response categories of Questions 1 and 3. The level of activity time derived from the interview increased in the order of the response categories of questions for both men and women. Of the subjects who reported the least activity on the response to Question 1, 5% of the men and 3% of the women had average activity times of at least 5 hours per week according to the interview. These subjects accounted for 3.3% of all men and 2.2% of all women in the study. Conversely, of the subjects who reported the most activity on the response to Question 1, 20% of the men and 21% of the women had average activity times of less than 1 hour per week. These subjects accounted for 1.9% of all men and 1.6% of all women in the study. Thus, these extreme misclassifications accounted for almost 20% of the most active category, but only accounted for a very small percentage of the total group of respondents.

**Table 5.** Distributions of time spent on sports and physical exercise, derived from the interview, for each response category of Questions 1 and 3.

	Men N (%)	Time (min/week)				Women N (%)	Time (min/week)			
	
		25%ile	Median	75%ile	P*		25%ile	Median	75%ile	P*
Question 1 : time										
little	481 (65.1)	0	0	30	0.0001	655 (66.1)	0	0	17	0.0001
1-2hr/week	123 (16.6)	24	70	130		178 (18.0)	23	74	168	
3-4hr/week	66 ( 8.9)	35	149	240		82 ( 8.3)	54	140	223	
>=5hr/week	69 ( 9.3)	109	295	518		76 ( 7.7)	72	244	420	

Question 3 : frequency										
seldom	407 (55.1)	0	0	25	0.0001	614 (62.0)	0	0	13	0.0001
sometimes	182 (24.6)	2	38	114		152 (15.3)	6	36	154	
about once/week	55 ( 7.4)	53	123	240		89 ( 9.0)	40	83	172	
>=twice /week	95 (12.9)	50	220	461		136 (13.7)	86	180	348	

* Kruskal-Wallis test.

The weighted kappa coefficients calculated from data obtained from the first and second surveys are shown in Table 6. The coefficients of Questions 1 and 3, classified according to sex and age, ranged from 0.39 to 0.56, showing moderate reliability; those of Question 2 ranged from 0.25 to 0.39, showing fair reliability. Thus, the reliability of the walking question was weaker than that of the sports and physical exercise questions.

**Table 6.** Weighted kappa coefficients (95% confidence interval) of Questions 1-3 at an approximately 1-year interval.

	All	Age group (yr)		Interval between surveys (month)	
	
		40-59	60-79	11.5-12.4	12.5-15.9
Men					
Question 1 : time spent on sports and physical exercise	0.45 (0.37-0.53)	0.39 (0.23-0.54)	0.45 (0.35-0.54)	0.44 (0.34-0.54)	0.46 (0.31-0.61)
	n=416	n=166	n=250	n=302	n=114
Question 2 : daily walking time	0.32 (0.24-0.40)	0.36 (0.24-0.48)	0.30 (0.20-0.40)	0.34 (0.26-0.43)	0.20 (0.053-0.35)
	n=409	n=162	n=247	n=295	n=114
Question 3 : frequency of sports and physical exercise	0.50 (0.42-0.58)	0.52 (0.39-0.64)	0.48 (0.38-0.58)	0.48 (0.39-0.58)	0.53 (0.39-0.67)
	n=387	n=160	n=227	n=275	n=112

Women					
Question 1 : time spent on sports and physical exercise	0.40 (0.33-0.46)	0.40 (0.30-0.50)	0.39 (0.31-0.47)	0.40 (0.33-0.48)	0.37 (0.27-0.48)
	n=636	n=278	n=358	n=452	n=184
Question 2 : daily walking time	0.31 (0.25-0.38)	0.39 (0.29-0.48)	0.25 (0.17-0.33)	0.30 (0.22-0.37)	0.36 (0.25-0.46)
	n=626	n=274	n=352	n=440	n=186
Question 3 : frequency of sports and physical exercise	0.51 (0.44-0.57)	0.56 (0.47-0.65)	0.47 (0.38-0.55)	0.55 (0.47-0.63)	0.40 (0.30-0.51)
	n=588	n=265	n=323	n=409	n=179

Total number of subjects varied by question because of missing values.

The reliability coefficients did not vary substantially when they were calculated for subjects who were surveyed at an interval very close to 12 months with both surveys conducted in the same season. However, for those who were surveyed at an interval of 12.5 to 15.9 months, there was some variability, with the coefficient of Question 2 lower for men and the coefficient of Question 3 lower for women.

## DISCUSSION

The validity of a survey instrument is commonly defined as the extent to which it measures what it is intended to measure^21^^)^. The phenomenon that a survey is intended to measure must be clearly defined for the subjects in a self-administered questionnaire survey. When the phenomenon being measured is sports and physical exercise (“undou”), the definition must include the purpose, type, and intensity of the activity as well as the reference period of interest^23^^, ^^24^^)^. The questions on the JACC questionnaire were not accompanied by such information, which might have led to misunderstandings. Furthermore, although we intended to focus on activity over a long reference period, such as 1 year, the subject’s response might have been biased by the subjects recalling their most recent activities and attributing that frequency to the entire year.

Once the definition of “undou” (physical exercise) was determined, we compared responses to questions on the JACC questionnaire with the criterion measure derived from the interviewing method used in the Japan Lifestyle Monitoring Study. In this interview, after asking a question very similar to Question 1, the interviewer added several questions to discern which activities to exclude because they were outside of the stated definition and which activities to include because they were otherwise being overlooked. The validity correlation coefficients of the responses to Questions 1 and 3 with activity time or LTPA score derived from the interview ranged from 0.43 to 0.60. These coefficients were better than those (r = 0.25-0.31) of responses to a single-item question about usual physical activity correlated with an energy expenditure index obtained from another detailed question set, reported by Weiss et al^12^^)^. Our correlation results suggest moderate validity of Questions 1 and 3 relative to indices which reflect the act of engaging in sports and physical exercise over a year, easing concerns that those questions might have caused misunderstanding.

The correlations of Question 1 or 3 with the LTPA score were almost the same as the correlations with activity time derived from the interview. The reason for this is that the LTPA score, an index of energy expenditure, showed an extremely high correlation with activity time (Spearman’s r = 0.96 when activity time exceeded 0).

The criterion validity is generally estimated using the criterion measure currently assumed to be the gold standard. Whether the criterion measure derived from the interviewing method in the present study should be regarded as the gold standard is arguable. However, the criterion measure need not be the gold standard, but it does need to be an established and presumably better measure than the ones selected for comparison^12^^)^. We assumed that the interviewing method is more accurate than the self-administered, single-item questions, and also that it has the advantages of not altering individual behavior, not being intrusive, and being practical in a large-scale validation study, unlike other methods^25^^)^. However, there is the disadvantage of introducing the same source of error, memory, in both the self-administered questionnaire and interview^26^^)^.Therefore, the errors of both methods might remain correlated, and the validity we assessed in the present study might be higher than the validity assessed by criterion measures in which data are objectively recorded or sensor-monitored. In spite of this possible study limitation, we determined the validity of measuring physical activity using simple, single-item questions, relative to measuring physical activity using a detailed interview requiring more time and effort from both the participants and the interviewers.

We did not conduct a validation study of Question 2, because there was no established interviewing method that could be regarded as a criterion measure (gold standard); i.e., a method believed to be more accurate than Question 2 and practical for measuring walking time averaged over a relatively long reference period. However, it would be worthwhile to assess the validity of a single-item question about walking by comparing responses to a self-administered questionnaire with results assessed by criterion measures in which data are objectively measured, such as data obtained with a pedometer.

The reliability coefficients of Questions 1, 2, and 3 at nearly a 1-year interval, classified according to sex and age, ranged from 0.25 to 0.56. Reliability is the extent to which repeated measurements of a stable phenomenon get similar results^27^^)^, but physical activity is not necessarily a stable phenomenon, particularly over a long time. The reliability assessed in the present study reflects both variation in response to the questions and true changes in physical activity over 1 year. The results of our study suggested that the long-term stability of physical activity assessed by these questions, including the performance of measurements, is fair to moderate.

Test-retest reliability is generally believed to become lower as the interval between tests becomes longer. However, Tsubono et al. reported that reliability of a self-administered food frequency questionnaire tended to be lower when the questionnaires were administered in different seasons at a 5-month interval than when they were administered in the same season at a 1-year interval, in their multiple-interval reliability study^28^^)^. Given these results, it seems reasonable to speculate that seasonal variation also affects reliability of physical activity surveys. In the present study, some reliability coefficients for subjects who were surveyed at intervals of 12.5 to 15.9 months were apparently lower than those for subjects who were surveyed at intervals very close to 12 months. These results may indicate the effects of seasonal variations. However, these pairs of coefficients, obtained from repeated surveys conducted in different seasons and the same season, were measured for different subgroups, and the difference between the two coefficients did not necessarily reflect within-person variations. Further investigations will be required to clarify the effects of seasonal variations on the reliability of these questions.

In the present study population, the responses to Questions 1, 2, and 3 showed biased distributions. We thought the number of subjects in the least frequent response category was large enough to overcome effects of chance and provide appropriate evidence of validity and reliability, such as a significant level of criterion measure in each response category and meaningful coefficients. The sample size of the present study was sufficiently large for 40- to 79-year-old people.

Most of the subjects in the present study were selected from JACC baseline study fields, and the biased distributions of their responses to Questions 1 and 2 were similar to those found among almost 80,000 people 40-79 years old in the JACC baseline survey^29^^)^. The validity and reliability observed in the present study is considered generalizable to those people who participated in the JACC baseline survey.

In summary, the validity of Questions 1 and 3 was moderate, and the reliability of Questions 1, 2 and 3 at a 1-year interval was fair to moderate. We suggest that measuring physical activity level with these single-item questions may be appropriate for establishing baseline data that reflects long-term physical activity in a large-scale cohort study targeting lifestyle-related diseases.
